# Corallicolids: The elusive coral-infecting apicomplexans

**DOI:** 10.1371/journal.ppat.1009845

**Published:** 2021-09-16

**Authors:** Patrick J. Keeling, Varsha Mathur, Waldan K. Kwong

**Affiliations:** Department of Botany, University of British Columbia, Vancouver, Canada; University of Wisconsin Medical School, UNITED STATES

Coral reefs are the poster child for ocean biodiversity—a marine equivalent of the terrestrial tropical rainforest in their scientific importance and public perception. Compared with most of the ocean, coral reefs harbour an incredible variety of biodiversity, and this diversity is also fairly well studied so that many of the basic interactions making up the ecosystem are understood at some level. The foundation of the system is obviously coral, particularly reef-building, scleractinian corals. The calcium carbonate skeletons secreted by these animals are literally the bedrock of the reef and create habitats that other reef creatures depend upon [1]. At this macroscopic scale, the importance of coral is intuitive to us. However, it’s becoming clear that at another, more abstract level, coral reefs are also supported by diverse microscopic communities, some of which play a direct role in coral health and collectively serve as a different kind of ecological foundation for the reef [2].

Coral reefs are also a model for understanding symbiosis. The large stony coral you see when diving or snorkeling (Fig 1) is actually a colony of heterotrophic animals, yet the small prey they filter from seawater is insufficient for the massive energy expenditure required to build reefs. For that, they have formed a symbiotic partnership with a lineage of photosynthetic dinoflagellates, the Symbiodiniaceae [3], which harness light energy to fix carbon and feed calcium carbonate production. This coral–microbe symbiosis is clearly important to reef building and coral health, but we are increasingly seeing that it is only one part of the story. Coral is actually home to rich microbial communities that vary with host, location, depth, season, and coral health [4–9]. Coral health and disease is a topic of growing concern: As corals are challenged by systemic problems relating to warming seas, overfishing, and ocean acidification, the spread of disease has emerged as one important factor in their reaction to these challenges [10,11]. Several important coral pathogens have been identified [12], and, recently, a new family of coral-infecting microbe has been discovered by a most unusual means—not by the study of disease or pathology, but as a chance discovery from ecological research [13–15]. These new intracellular parasites are members of the Apicomplexa, well known as parasitic agents of humans and other animals (e.g., *Toxoplasma*, *Cryptosporidium*, or the malaria agent *Plasmodium*) and are now called Corallicolida [16] (Fig 2).

**Fig 1:**
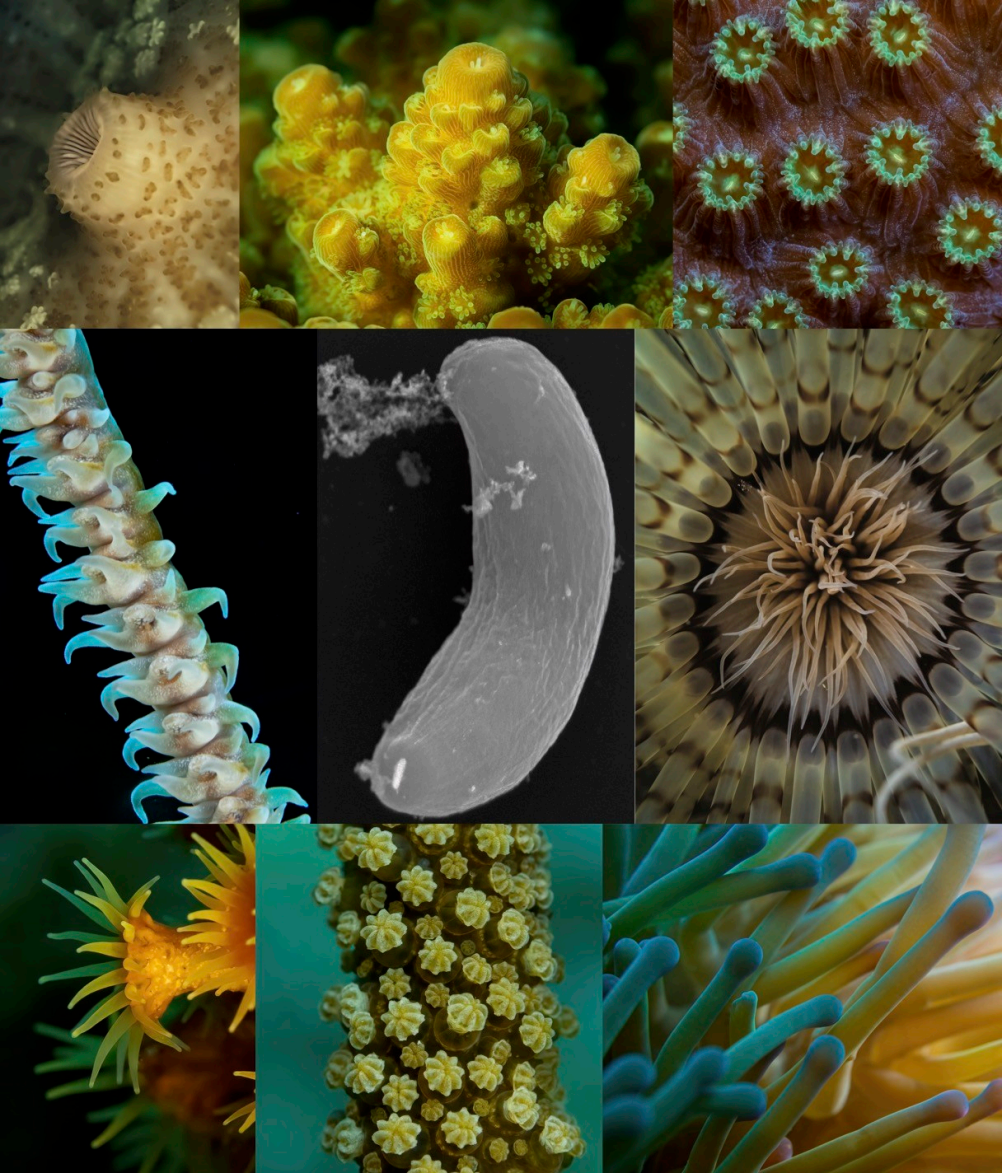
Corallicolids and their hosts. A variety of anthozoan animals surrounding a corallicolid (centre: SEM of *Anthozoaphila gnarlus*). Clockwise from top left: *Rhodactis* (the type host of *Corallicola aquarius*), the complex scleractinian *Acropora*, the robust scleractinian *Orbacella*, the tube-dwelling anemone *Pachycerianthus*, the actiniarian anemone *Condylactis*, the gorgonian *Eunica*, the zoanthid *Parazoanthus*, and the black coral *Cirrhipathes*.

**Fig 2:**
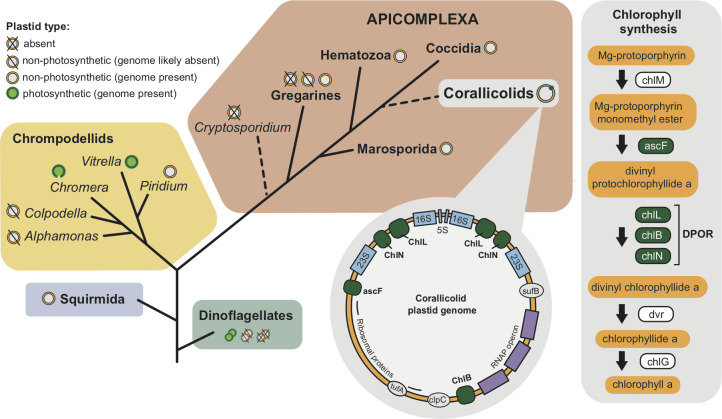
Phylogeny of the Apicomplexa and their close relatives depicting the putative phylogenetic position of the corallicolids and their unique plastid. A summary of the distribution of plastids, photosynthesis, and plastid genomes is plotted on the tree (the *Chromera* plastid genome is shown linear, and the dinoflagellate plastid genomes are fragmented into many small chromosomes). The presence of 4 genes involved in chlorophyll synthesis (*acsF*, *chlL*, *chlN*, and *chlB*) on the corallicolid plastid genome are shown in dark green on a simplified plastid map. The putative function of these enzymes in the chlorophyll synthesis pathway is presented. All other enzymes in this pathway (*chlM*, *chlG*, and *dvr*) are ancestrally plastid-targeted proteins that are encoded by nuclear genes (so their presence in corallicolids remains to be tested as genomic data are not yet available). DPOR, dark-operative protochlorophyllide reductase; RNAP, RNA polymerase.

## How do you discover a eukaryotic parasite from bacterial ecological data?

Microbial ecology has long relied on molecular data to survey and distinguish between the vast diversity of microbes in an environment. Decades of study has generated a huge database of environmental sequences, but a disproportionate amount of these data are from bacteria and archaea: In ecology, as in most areas of microbiology, the microbial eukaryotes are relatively unloved and understudied! In this case, ignoring eukaryotes had consequences. Identifying environmental sequence data requires comparisons to accurately curated reference sequences, and these often lack a broad representation of eukaryotes—or their organelles [17]. Mitochondria and plastid organelles are derived from ancient bacterial endosymbionts, and most retain a relict genome that encodes the small subunit rRNA gene (SSU rRNA or 16S rRNA) used in ecological surveys. As a result, millions of eukaryotic plastids are represented in rRNA surveys but are often either overlooked, filtered out as contaminants, or misattributed to unknown or “novel” phyla of bacteria. In the case of coral-associated 16S rRNA surveys, a detailed phylogenetic analysis of these plastid “contaminants” showed that most of them fell within known groups of algae, with one major exception: thousands of sequences that branched in several subgroups as sister to the plastids of apicomplexan parasites [14,18]. Moreover, virtually, all of these apicomplexan-related lineages (ARLs) came from studies of coral or other substrates in the reef.

As intriguing as these findings were, they raised many more questions because ARLs were known only as plastid 16S rRNA gene sequences that revealed little detail about the identity of these organisms, their association with coral, or their biological traits and characteristics. A single similarly mysterious apicomplexan lineage called “Genotype-N” had also been observed in nuclear 18S surveys of coral [13], and this genetic marker was subsequently used to track its distribution and transmission [19–21]. A single species of coral-infecting apicomplexan had also been formally described as *Gemmocystis cylindrus* from histological samples [22].These disparate data all showed that apicomplexans infected coral, yet they were also all virtually incomparable to one another, except that the phylogenetic positions of ARL-V and type-N were inconsistent. Were these all the same organism? Were ARLs deep branching beneficial photosynthetic symbionts (like Symbiodiniaceae), or were they intracellular parasites (like other apicomplexans)? Answering these basic questions required finding a needle in a haystack: ARLs were widespread, but not abundant; they were found globally in a diverse variety of coral species, but appeared to be strictly intracellular (or tightly host associated) and not free in the environment [18]. This needle was found, not from sunny tropical islands, but from aquarium corals sourced from an urban pet shop [15]. The first observations of corallicolid cells linked by in situ hybridization to both nuclear and plastid rRNA sequences confirmed that they were found intracellularly within the mesenterial filaments of coral and corresponded to both ARL and type-N environmental sequences. Furthermore, corallicolid cells were found to be colourless, which would generally not be expected of a photobiont. Sequencing of the complete plastid genome from the same sample verified this: The genome lacked all photosystems, and the plastid was therefore incapable of photosynthesis. Corallicolids are not photosynthetic symbionts.

## An ancient group of apicomplexans in an ancient group of animals?

The first molecular data from corallicolid plastid rRNA suggested that they were deep-branching sisters to apicomplexans [14]. This was an eye-catching possibility, since corals are also an ancient group of animals [23], and the closest known photosynthetic relatives of apicomplexans (*Chromera* and *Vitrella*) are also associated with corals [24,25] (Fig 2). Soon after the discovery of plastids in *Plasmodium* and *Toxoplasma*, it was proposed that apicomplexan parasitism might have arisen through an intermediate stage of photosymbiosis [26]. These new discoveries painted a vivid picture where coral played the role of the host—betrayed by their apicomplexan endosymbionts who lost photosynthesis and became parasites. But more data have altered and complicated this simple picture. Specifically, phylogenies based on mitochondrial and nuclear sequences place corallicolids not as sisters to all other apicomplexans, but as a unique subgroup of the large and diverse coccidian lineage (Fig 2) [15]. These phylogenies are moderately supported but importantly exclude branching of the corallicolid lineage before that of the early diverging gregarine apicomplexans. Plastid phylogenies continue to show corallicolids branching earlier, but analyses of individual plastid genes show inconsistency in the phylogenetic signal and a susceptibility to artefacts arising from accelerated substitution rates [27,28]. Overall, the plastid rRNA continue to be the best sampled gene for environmental inferences, but the plastid-encoded genes remain a poor choice for phylogenetic inferences. Conversely, the conclusion that corallicolids are closely related to coccidian apicomplexans (that include medically important apicomplexans such as *Toxoplasma gondii*) is likely, but requires more data: We still lack a large nuclear genomic survey from any corallicolid, and we also lack representation from other potentially key apicomplexan groups. For instance, the Adelerina apicomplexans are also sometimes hypothesised to be related to coccidia [29]; however, they, too, lack any representative nuclear genomic-level data. A clearer picture of the origin of corallicolids is going to require more data not only from the coral parasites themselves, but also from a number of other overlooked apicomplexan lineages.

## Do corallicolids infect anything else?

This sounds like a simple question but confoundingly depends on how you define “coral.” When we think of coral, we usually mean the Scleractinia, a monophyletic subgroup of the Anthozoa that includes the familiar stony reef-building corals. Yet, the Anthozoa (Fig 1) also includes a wide variety of mostly marine animals, and the terms “coral” and “anemone” are applied to multiple subgroups scattered across the anthozoan tree. For example, black corals are distinct from stony corals, and the same subgroup (hexacorals) also includes sea anemones, corallimorphs, and zoanthids. Octocorals (e.g., gorgonians, fan corals, and sea pens) are a more distant subgroup of Anthozoa and include animals commonly referred to as “soft corals.” Tube-dwelling anemones are a third major subgroup and are distinct from other anemones. But, luckily, we can ignore some of this confusing terminology, because corallicolids have now been found to infect most anthozoan subgroups examined to date, whether or not we call them corals, or anemones, or anything else.

Collectively, corallicolids infect a wide variety of anthozoans, but what about individual corallicolid species—what is their host range? We can only infer this from environmental sequencing data, which were not designed to answer such questions and lack the resolution for strain or even species level distinctions, but the answer appears to be complex. Some sequence variants are found in multiple anthozoan hosts, but the same host species can also harbour different corallicolids, both suggesting a broad host range and limited discrepancy between hosts [15,16,18,30]. Conversely, other corallicolid lineages have been found to be associated with a single host or host clade, suggesting host specificity in these lineages, and, perhaps, even coevolution [16,30]. Resolving these questions will require sampling using sequence markers that can resolve fine-scale differences between closely related corallicolid populations and sampling from a large number of both closely and distantly related hosts (including multiple individuals of the same species). Key questions will be whether any particular corallicolid is a generalist infecting many hosts, whether any corallicolid lineages have cospeciated with specific hosts, and whether there are any geographical patterns to corallicolid populations.

An even larger question is whether any corallicolids infect nonanthozoan hosts. Other cnidarians (e.g., hydroids and various “jellies”) are a likely place to look, but, to date, corallicolid infections have not been documented in these hosts (including large, coral-like colonial hydroids such as lace corals or fire corals). One major finding, however, comes unexpectedly from bony fish. Haemogregarines are a common apicomplexan parasite in fish blood, but recent molecular taxonomic work showed that haemogregarines from many marine fish were not haemogregarines at all: Instead, these parasites formed a distinct lineage distant to other haemogregarines described from freshwater fish [31]. In fact, this lineage of fish blood parasites is closely related to corallicolids. This is a very exciting finding, suggesting that they and corallicolids might form a larger marine lineage of parasites, but beyond their life cycle [32], we know little of these organisms or what traits they may share with their coral-infecting kin.

## Agents of coral disease?

Very little is known about corallicolid biology or their effects on the coral host. Given what we know from their apicomplexan relatives, it is unlikely that corallicolids are greatly beneficial to the coral, but there is also no evidence they are particularly pathogenic. In some coral species, the relationship between corallicolids and corals begins as early as the host larval stage [21]. Preliminary analyses of environmental sequence data have been compared with the observed health of the host tissue and did not find a strong correlation between the presence of corallicolids and recognisable disease [18,33]. However, the situation is undoubtedly more complex. First, environmental sequence surveys are a snapshot of what exists in the sample at that time and do not take into account more subtle relationships like opportunistic pathogenesis: Corallicolids may be benign under favourable environmental conditions, but pathogenic to hosts under stress. Similarly, corallicolids with broad host ranges may be harmless to some hosts, but harmful to others. A similar situation has been found in a coral-associated bacteria, *Aquarickettsia*. Following from its initial discovery through molecular surveys, its relationship with the coral has been found to be broad and context dependent [34,35]. Overall, both the host and corallicolid lineages are phylogenetically diverse, and, as we learn more about their biology, we probably should expect a diverse spectrum of host–symbiont relationships.

## Chlorophyll without photosynthesis?

When the first corallicolid plastid genome was described, they were ruled out as being photosynthetic symbionts because the genome was completely devoid of photosystem genes common to all photosynthetic plastid genomes. However, the corallicolid plastid genome did contain 4 genes not found in other parasitic apicomplexan plastids, and these represented all 4 of the chlorophyll biosynthesis genes that would have been ancestral to apicomplexans (Fig 2) [15]. These genes were shown to be expressed and under more stringent purifying selection than most other genes in the plastid genome, thus strongly indicating that they maintain an essential function. It is hard to overemphasise how biochemically strange it is to retain chlorophyll but not photosynthesis: Harvesting photons to liberate high-energy electrons in the absence of photosystems for the controlled dissipation of that energy is biochemically akin to deliberately detonating a bomb inside your cell. Corallicolids lack chlorophyll autofluorescence and are certainly not visibly pigmented; therefore, they likely do not produce chlorophyll in large quantities, and it is unlikely to be complexed with reaction centres (genes for which are absent from the plastid), so its role in the cell must be unrelated to converting energy. What this role might be is currently a major question, but without even basic data on the biology of corallicolids, there is no clear answer. However, one intriguing new line of enquiry emerged recently from cryptomonad algae, where a nonphotosynthetic species was also found to retain some chlorophyll biosynthesis genes [36]. The function of these cryptomonad genes is also unknown, but perhaps these 2 cases hint at a cryptic function for the chlorophyll pathway, perhaps even one widely utilised in organisms transitioning from photosynthetic to nonphotosynthetic lifestyles.

Chlorophyll is a member of the porphyrin family of molecules, and several other products of this pathway serve various roles in the cell (e.g., heme, which is partially produced in the plastids of other apicomplexans) [37], so one possibility is that a chlorophyll precursor or variant is used in some as yet unidentified pathway. Another possibility is that chlorophyll is retained because of an unrecognised but ancient feedback inhibition pathway that regulates a more general step in porphyrin biosynthesis. These and many other possible explanations would lead to fascinating insights into potentially ancient biochemical networks that are harder to see in photosynthetic organisms where chlorophyll is more abundant. The technical challenges that we face in developing tools and resources to dig more deeply into corallicolids and their symbiosis with coral are substantial. In addition to more detailed surveys of their natural diversity and distribution, it is vital to generate genomic resources and culture techniques, both of which will bring us closer to understanding the unique biology of these elusive coral apicomplexans.
